# Therapeutic Targets in Amyotrophic Lateral Sclerosis: Focus on Ion Channels and Skeletal Muscle

**DOI:** 10.3390/cells11030415

**Published:** 2022-01-25

**Authors:** Nancy Tarantino, Ileana Canfora, Giulia Maria Camerino, Sabata Pierno

**Affiliations:** Department of Pharmacy-Drug Sciences, University of Bari Aldo Moro, 70125 Bari, Italy; nancy.tarantino@uniba.it (N.T.); ileana.canfora@uniba.it (I.C.); giuliamaria.camerino@uniba.it (G.M.C.)

**Keywords:** amyotrophic lateral sclerosis, skeletal muscle, ion channels, animal models, clinical trials, therapy

## Abstract

Amyotrophic Lateral Sclerosis is a neurodegenerative disease caused by progressive loss of motor neurons, which severely compromises skeletal muscle function. Evidence shows that muscle may act as a molecular powerhouse, whose final signals generate in patients a progressive loss of voluntary muscle function and weakness leading to paralysis. This pathology is the result of a complex cascade of events that involves a crosstalk among motor neurons, glia, and muscles, and evolves through the action of converging toxic mechanisms. In fact, mitochondrial dysfunction, which leads to oxidative stress, is one of the mechanisms causing cell death. It is a common denominator for the two existing forms of the disease: sporadic and familial. Other factors include excitotoxicity, inflammation, and protein aggregation. Currently, there are limited cures. The only approved drug for therapy is riluzole, that modestly prolongs survival, with edaravone now waiting for new clinical trial aimed to clarify its efficacy. Thus, there is a need of effective treatments to reverse the damage in this devastating pathology. Many drugs have been already tested in clinical trials and are currently under investigation. This review summarizes the already tested drugs aimed at restoring muscle-nerve cross-talk and on new treatment options targeting this tissue.

## 1. Introduction

Amyotrophic Lateral Sclerosis (ALS) is a progressive neurodegenerative disease. It is characterized by selective degeneration of upper and lower motor neurons (MN), which causes weakness, muscle wasting, and fasciculation. These symptoms suggest a strong involvement of skeletal muscle in the pathology. Among the causes of MN degeneration, the glutamate induced excitotoxicity plays a major role [1]. It was also shown that glial cell dysfunction contributes to the disease [2]. At the NMJ, alterations in perisynaptic Schwann cell (PSC), the glial cells placed at this synapse, can influence their ability to regulate NMJ stability, impairing compensatory reinnervation in ALS. In addition, PSC alteration compromises the supply of several trophic molecules important for muscle-nerve communication.

Affected individuals suffer from progressive paralysis and can die within 3 to 5 years after the onset of symptoms due to respiratory failure. ALS is sporadic (sALS) in about 90% of cases, and the remaining 10% are of genetic (fALS) origin, with a large subgroup carrying mutations in the superoxide dismutase enzyme (SOD1), a cell scavenger of superoxide anion symptoms [3]. Other mutations in several genes, such as ANG (angiogenin), DCTN1 (dynactin), TDP43 (TAR DNA-binding protein 43), FUS (protein Fused in Sarcoma), and C9orf72 (chromosome 9 open reading frame 72) were linked to familial forms of ALS [4]. In the last years, several animal models were generated by reproducing various mutations, with the aim of studying this pathology and the involvement of various tissues [5]. Actually, clinical symptoms and the pathogenic mechanisms were found to be the same in sporadic and familial cases of ALS.

Despite the fact that it was clinically described many years ago, the mechanisms underlying ALS pathogenesis are not yet fully understood. Studies in the animal models of ALS and in the patients reveal a plethora of alterations, such as an increase of glutamate-mediated excitotoxicity, oxidative stress, defective axonal transport, a dysregulation in autophagy and ubiquitin proteasome system, protein-misfolding events, mitochondrial impairment, and alteration of immune responses [6]. Thus, with the aim to better investigate the etiopathogenesis of the disease, the preclinical analysis of transgenic mouse models can be helpful and very important not only for studying the mechanisms leading to the tissue damage, but also to test new effective drugs to submit to clinical trials. The SOD1-G93A transgenic mouse model is the most studied because it recapitulates several aspects of the disease allowing the drafting of a clear hypothesis [6,7,8]. The genetic mutation is ubiquitously expressed and is linked to a toxic gain of function of the SOD1 enzyme, with the generation of free radicals that leads to cell injury and death. Additionally, the mutation induces conformational instability and misfolding of SOD1 protein, resulting in the formation of intracellular aggregates that inhibit normal proteasomal function, disrupting axonal transport and vital cellular functions. Additionally, the mutation in the transactive response DNA-binding protein43 (TARDBP gene, TDP43 protein) is a common cause of ALS [4,9,10]. To date, more than 40 TARDBP mutations are associated with the ALS phenotype. TDP43 was identified as a key component of the insoluble and ubiquitinated inclusions in the brain of ALS patients. Full length, wild type TDP43 is found to be aggregated in the vast majority of sALS and fALS patients. Studies in vivo and in vitro demonstrate that this protein is not particularly prone to aggregation by itself, unless it is highly overexpressed. Protein aggregates may arise as a consequence of improper folding of a mutant protein, but also following oxidative modifications of crucial proteins, defective chaperons, and protein degradation.

At present, there are neither clear biomarkers nor resolutive therapies or disease-modifying treatments for ALS. The only approved treatment option is riluzole, which is able to reduce the glutamate-induced excitotoxicity [11]. However, riluzole increases survival of patients by only a few months [12]. Thus, it is possible that different drugs acting at various levels and on various tissues may be useful in solving this multifactorial disease. Indeed, recently, the antioxidant edaravone was introduced in therapy because of its role as a disease-modifying agent [13]. Unfortunately, it was rapidly withdrawn from the European market and waiting for further efficacy studies.

## 2. Pathological Features and Skeletal Muscle Involvement

Skeletal muscle is now considered as an important target tissue involved in the pathogenesis of ALS, through activation of a retrograde signaling cascade that degrades affected motor neurons [14,15]. Skeletal muscle function is regulated by numerous factors, including satellite cells, cell metabolism, mitochondrial activity, and RNA processing. In ALS, these factors show various levels of dysregulation within skeletal muscle. Disease onset is accompanied with cramping and twitching, spasticity, severe muscle weakness, and atrophy in limbs and trunk. The pathology progresses to paralysis of voluntary muscles, including diaphragm. Muscle atrophy has been observed in both the classical mouse model SOD1-G93A and in the transgenic mouse model, in which the mutant SOD1 is expressed only in skeletal muscle (MLC/SOD1-G93A) and not in the neurons [16]. These mice also show reduced tetanic and specific force as well as NMJ abnormalities. This model was demonstrated to be useful in studying the involvement of skeletal muscle in the pathology. The appearance of early events before denervation were described in skeletal muscle of these animals (MLC/SOD), supporting the “dying back” hypothesis.

In this context, we investigated sarcolemma ion channels that play a crucial role in muscle function and are important to maintaining NMJ stability. Resting chloride conductance (gCl), sustained by the muscle ClC-1 channel, controls sarcolemma excitability [17,18]. Indeed, a reduction of ClC-1 channel activity and resting gCl generates myotonic-like symptoms [19,20,21]. Skeletal muscle potassium channels are important for skeletal muscle function since they are involved in cell excitability and metabolism. Interestingly, we found that macroscopic resting gCl was strongly reduced in SOD1-G93A mice as compared to wild-type (WT), and potassium conductance (gK) was significantly increased. In accord, patch clamp studies showed different activity of the KATP channels and an altered sensitivity to ATP. Consequently, sarcolemma excitability was increased. Additionally, in MLC/SOD1-G93A mice, we found a reduction of gCl which was restored by the in vitro application of chelerythrine, an inhibitor of protein kinase C (PKC), suggesting its involvement in the reduction of gCl [22]. Indeed, it is known that PKC is a regulatory protein of the ClC-1 activity, since it phosphorylates and closes the channel [23,24]. A multivariate statistical analysis of PCR data, using machine learning algorithms, identified some discriminant genes in these mice. Surprisingly, a modification of the expression of different ion channels in skeletal muscle was found. In particular, the expression of ClC-1, which is known to be the major chloride channel expressed in skeletal muscle, was reduced. This was accompanied by the increased expression of PKC, also involved in NMJ disruption [25,26]. We also showed that the expression of other genes were reduced in skeletal muscle, such as irisin, a pro-myogenic factor able to contrast denervation [27]. All these data demonstrate the involvement of skeletal muscle in the pathology.

**Figure 1:**
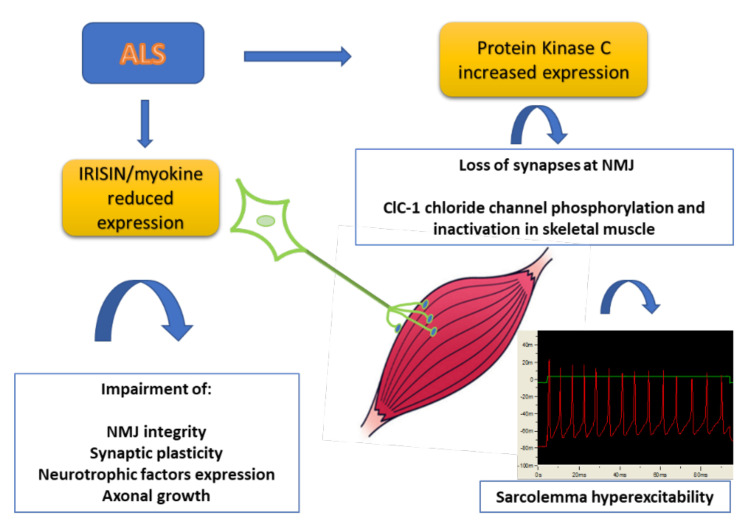
Scheme of the pathological events involving new biomarkers of ALS. Skeletal muscle is partially responsible for the MN decline and is in turn affected by denervation. The production of myokines is severely impaired in skeletal muscle with alteration of NMJ integrity and axonal growth. In addition, the increased expression of Protein kinase C can be responsible for the loss of synapses at NMJ and impairment of skeletal muscle excitability and function (see text for details).

Additional support to the important role of skeletal muscle was provided by the observation that the postnatal development in the absence of skeletal muscle results in the sequential ablation of motor neurons from the spinal cord to the brain [28], demonstrating that the nervous tissue development is coupled to skeletal myogenesis. Moreover, the expression of mutant SOD1 in motor neurons alone is not sufficient to cause ALS-like symptoms in mice [29,30,31]. Based on these results, it cannot be excluded that skeletal muscle is involved in ALS etiopathogenesis at the same time. Thus, skeletal muscle can be an attractive target of therapy, because drug specifically affecting skeletal muscle can be useful to ameliorate NMJ and MN function and can be used together with current therapies (Figure 1).

## 3. Current Clinical Trials by Using Drugs Targeting Skeletal Muscle

To date, riluzole is the only available drug for ALS therapy from 1995. This drug is neuroprotective, reducing the excitotoxic effect of excessive glutamate [32] and inhibiting voltage-dependent sodium channels [33,34]. Recently, the free radical scavenger, edaravone, was evaluated in a phase 3 randomized study and then introduced in therapy, but rapidly withdrawn in Europe and waiting for further efficacy studies. Indeed, this trial needs to be re-evaluated because of the small study size, the short study duration, and the lack of proven data on survival [35]. Yet, the anti-inflammatory and neuroprotective tyrosine kinase inhibitor, masitinib, showed new possibilities for cure [36]. Indeed, in a randomized controlled trial, when added to riluzole, it showed slight positive effects to be further evaluated.

During the last 10 years, the number of clinical studies focusing on ALS has grown exponentially. Potential therapeutic agents with different mechanisms of action have been tested with the aim to target one or more of the pathological aspects of this multifactorial disease (i.e., oxidative stress, inflammation, mitochondria, and neurotrophic factors deficit). Unfortunately, many of the tested compounds failed to induce some benefits on disease progression or survival. It is important to underline that there are many potential reasons for this failure. Indeed, clinical trial design is complicated by the low number of patients, different ALS patient subpopulations, late stage of initiation of clinical trial, and difficulty in the achievement of clinically relevant primary outcome measures. Moreover, the heterogeneity of the pathogenic mechanisms and the involvement of different tissues requires pharmacological multi-target strategies. For these reasons, it can be useful to search for more relevant outcome measures and new biomarkers of disease progression for trial design. In this context, skeletal muscle can be analyzed as an important drug target. Indeed, different studies show that the restoration of skeletal muscle function can have a possible neuroprotective role in this pathology, a phenomenon called saving-back [22,37]. Thus, drugs targeting skeletal muscle can also be useful for preserving NMJ integrity and delaying MN impairment. Here, we analyzed the past and current clinical trials aimed at targeting skeletal muscle and NMJ as useful therapeutic opportunities (ClinicalTrials.gov, accessed on 17 January 2022).

Pharmacological interventions often alleviate symptoms. As shown in Table 1, different drugs are categorized in a class of compounds able to affect the various pathological aspects. Drugs described to relieve spasticity and muscle cramps are baclofen, cannabinoids, botulin toxin, carbamazepine, or mexiletine, as well as magnesium supplements. Mitochondrial abnormalities described in skeletal muscle from ALS patients have been detected frequently in aggregates adjacent to the sarcolemma [38]. Accumulation of reactive oxygen species (ROS) were observed in skeletal muscle of ALS patients and SOD1-G93A mice [39]. These mice also showed an increase in cyclophilin D expression, which promotes the opening of the mitochondrial permeability transition pore (mPTP), increasing mitochondria membrane depolarization and further generation of ROS [39,40]. Indeed, the genetic deletion of cyclophilin D may delay disease onset and extend survival. Therefore, different drugs have been tested in clinical trials that aimed to ameliorate mitochondrial function and ATP production (Table 1). In particular, olesoxime, a cholesterol like compound that was already proposed for other invalidating pathologies, such as SMA [41]; and dexpramipexole, with neuroprotective properties by direct effects on mitochondria and stabilization of the proton gradients needed for ATP production [42]. Among antioxidant compounds, as an alternative to edaravone, creatine supplementation was tested in clinical trial without clear benefits [43]. Autophagy stimulators were tested, with the aim to recycle damaged cytoplasmic constituents and protein aggregates in skeletal muscle of ALS patients [44]. However, some authors claim that a great inhibition of mTOR can be detrimental in ALS condition, due to possible accumulation of cytoplasmic autophagosomes. In contrast, other authors describe a slight amelioration of symptoms [45]. To clarify this discrepancy, new clinical trials are ongoing [46]. The supplementation of trophic factors, such insulin-like growth factor 1 (IGF1) and growth hormone (GH), are able to stimulate growth and development of myogenesis through increase of protein synthesis. The up-regulation of IGF1 in muscle can improve hindlimb muscle strength and motor neuron survival. It was described that muscle-restricted expression of insulin-like growth factor type 1 (IGF-1) isoform maintained muscle integrity and enhanced satellite cell activity in SOD1-G93A transgenic mice, inducing calcineurin-mediated regenerative pathways [14,47]. However, in a clinical trial, subcutaneous recombinant human IGF1 administration did not corroborate these results, failing to detect changes in muscle strength or function [48,49]. Additionally, GH was ineffective. Recently, a new combination of two small molecules, sodium phenylbutyrate and tauroursodeoxycholic acid (TUDCA), seems to be promising in the restoration of muscle and neuronal function and increasing survival in ALS patients. These drugs show antioxidant, antiapoptotic, and neuroprotective effects in preclinical studies. Sodium phenylbutyrate helps proteins maintain their normal conformation, preventing aggregation. TUDCA improves mitochondrial energy production and endoplasmic reticulum normal function in cells [50]. Among drugs acting on skeletal muscle proteins, reldesemtiv is a fast skeletal muscle troponin activator. It is a small molecule designed to slow the release of calcium, improving muscle function and movement. This drug is more potent than other skeletal muscle activators, such as tirasemtiv, with the advantage of lower doses. Additionally, because reldesemtiv does not cross the blood-brain barrier, it should cause fewer significant side effects than tirasemtiv. Additionally, levosimendan, by sensitizing the skeletal muscle to calcium signaling, helps it to contract more easily. Skeletal muscle defect in ALS involves acetylcholine receptors (AChRs). Thus, endocannabinoid palmitoylethanolamide (PEA) can promote AChRs currents in ALS muscle [51]. Additionally, pimozide was tested in a clinical study based on its ability to inhibit the T-type Ca2+ channel and to promote beneficial effects at neuromuscular junctions (NMJs) transmission. In the past, it was shown to be unable to induce significant results [52]. However, new clinical trials are recruiting, based on positive preclinical and pilot studies showing an effect at doses lower than that used for other therapeutic indications [53]. Moreover, new pimozide derivatives are currently studied as promising drugs [54]. Old clinical studies have evaluated sport therapy as a possible beneficial measure. Physical therapy is supposed to improve the overall quality of life in patients with ALS. However, the studies that have been conducted to date were too small to determine the benefits of exercises for ALS patients. This therapy can help to reduce the frequency and intensity of muscle cramps, and to prevent pain and stiffness. Studies using ALS animal models have shown that the animals benefit from moderate exercise, but intense exercise causes an acceleration of weakness [55].

It should be underlined that other new clinical trials are based on genetic approaches. Antisense oligonucleotides (ASOs) are current drugs in development for the different genetic forms of the disease. ASO are short single stranded nucleotide sequences that bind mRNA to modulate gene expression. Although promising in preclinical models of ALS caused by SOD1 mutations and C9orf72 repeat expansions [56,57,58,59], they need long-time studies and elevated number of patients. It should be considered that these therapies often require administration by invasive routes (i.e., intrathecal or intracerebral) to reach the central nervous system (CNS) since they are unable to cross the blood-brain barrier (BBB) and can develop cytotoxicity. Moreover, these therapies are limited in the sporadic forms that represent the major percent of ALS forms. Recently, encouraging results have been reached using tofersen during phase 2 clinical trial in ALS patients with SOD1 mutation [60]. By reducing mutated SOD1 level, the drug showed to increase muscle force. Thus, despite the risk, antisense technology merits further investigation. Additionally, pyrimethamine was found to produce a significant reduction in total CSF SOD1 protein content in patients with ALS caused by different SOD1 mutations. Although the mechanism was not clear, pyrimethamine was found to be safe and well tolerated in ALS patients [61]. Further long-term studies are warranted to assess clinical efficacy. Moreover, clinical trials using stem cells are ongoing, although the effects on disease progression are not yet documented. For instance, stem cells programmed to secrete neurotrophic factors (NTFs) were planned to promote growth and survival of muscle and nerve cells [62]. Particular attention should be also taken with new technologies using synthetic microRNA anti-SOD1 (by adeno-associated virus), which are able to target and degrade SOD1 messenger RNA, thereby suppressing the expression of the gene in the spinal cord and slowing the progression of the disease. Several preclinical [63,64] and clinical studies [65] have shown the first positive results.

As known, alterations in multiple cell types act synergistically to exacerbate the disease [3], thus, it can be useful to control different pathways when possible.

**Table 1:** Drugs are grouped based on their ability to affect pathogenic mechanism involved in the modification of skeletal muscle function and motor neuron health.

Class of Drug	Drug/Agent	Mechanism of Action	Trial Number	Bibliography
Mitochondria protectants	Olesoxime	mitochondrial permeability and transition pore modulation	NCT01285583/ NCT00868166 (phase 3)	[66]
	Dexpramipexole	mitochondrial function enhancement	NCT01281189 (phase 3)	[42]
	Coenzime Q10	mitochondrial cofactor	NCT00243932	[67]
	Tamoxifen	protease and autophagy enhancement	NCT02166944 (phase 2)	
	Creatine	energy production stimulation and oxidative stress response activation	NCT00070993 (phase 2)	[43]
Muscle metabolism protectants	TUDCA + Sodium phenyl butyrate	skeletal muscle and nervous tissue protection	NCT03127514 (phase 3)	[50]
	IGF-1	anabolic pathways stimulation	NCT00035815 (phase 3)	[49]
	GH	anabolic pathways stimulation	NCT00635960	[68]
Fast skeletal muscle troponin activator	Tirasemtiv	contraction stimulation	NCT02496767 (phase 3)	[69]
	Reldesemtiv	contraction stimulation	NCT03160898 (phase 2)	[70]
Modulators of ion channels and excitability	Ezogabine/Retigabine	K+ channels activation, hyperexcitability inhibition	NCT02450552 (phase 2)	[71]
	Mexiletine	Na+ channel inhibition	NCT01811355 (phase 4)	[72]
	Dronabinol	TRP channels modulation and cramps relieve	NCT00812851 (not applicable)	
	Levosimendan	Ca++ sensitization K+ opening	NCT03505021 (phase 3)	[73]
Modulators of NMJ function	Endocannabinoid palmitoyl-ethanolamide (PEA)	stimulation of AChR expression and activity	NCT02645461 (not applicable)	[51]
	Pimozide	NMJ stabilization	NCT03272503	[52,53]
Muscle proteostasis	Rapamycin	stimulation of proteins degradation	NCT03359538 (phase 2)	[46]
	Colchicine	autophagy activation	NCT03693781 (phase 2)	[74]
Other mechanisms	1-(beta-D-Ribofuranosyl) nicotinamide chloride and 3,5-Dimethoxy-4′-hydroxy-trans-stilbene	NAD+ level increase and support of sirtuin activity	NCT03489200 (not applicable)	[75]
	Clenbuterol	motor function improvement	NCT04245709 (phase 2)	
	Ozanezumab	monoclonal antibody that targets neurite outgrowth inhibitor	NCT01753076 (phase2)	[76]
	Acthar gel	stimulation of steroids production and regulation of inflammation in skeletal muscle	NCT03068754	[77]
	Sport therapy	muscle metabolism	NCT02548663	[78,79]

TRP: Transient Receptor Potential channels; AChR: Acetylcholine Receptor.

## 4. Preclinical Studies and Proposed Drugs Able to Restore Skeletal Muscle Function

The development of novel therapeutic strategies targeting the skeletal muscle were planned to slow down the onset and progression of this disease [80] based on significant preclinical studies. Here, we report some examples of drugs preclinically tested to restore skeletal muscle function in ALS animal models and were also useful for improving MN performance (Table 2).

Different preclinical studies were performed in consideration of the modifications of trophic factors. For instance, it was found that glial cell-derived neurotrophic factor (GDNF) increased survival through a beneficial effect on the NMJ, since it ameliorates the nerve sprouting ability [81] and MN survival [82]. Since vascular endothelial growth factor (VEGF) promotes angiogenesis and neuronal survival [83], the VEGF supplementation in skeletal muscle of SOD1-G93A mice had positive effects on ALS symptoms [84]. Additionally, neuregulin 1 (NRG1), by activating cell survival pathways, protects NMJ and acetylcholine receptors from decline in SOD1-G93A mice [85]. In line with NMJs preservation, treated mice had better neuromuscular and motor functions [86]. Insulin-like growth factor 1 (IGF-1) is an anabolic compound that promotes satellite cell proliferation and muscle hypertrophy [4]. Muscle-directed gene therapy of IGF-1 in ALS models promotes survival in SOD1-G93A mice and ameliorates MN function [14,87]. Additionally, creatine is an important amino acid of skeletal muscle, proposed in preclinical and clinical studies to compensate energy depletion. This is correlated with a slower progression of the disease in ALS mouse models. Indeed, creatine supplementation ameliorated motor performance and extended survival in SOD1-G93A mice, protected neurons, and reduced the extent of oxidative damage. Due to these observations, supplementation of creatine was evaluated in two clinical trials, unfortunately without significant effects [88]. The anabolic-androgenic steroid (AAS) nandrolone protects mitochondria, reduces muscle atrophy, and supports diaphragm muscle function but does not prevent muscle denervation [89]. Additionally, the AMP-activated protein kinase (AMPK) had a beneficial role in skeletal muscle; indeed, the removal of AMPK from wild-type mice promotes modification of function, as observed in SOD1 mice [90]. AMPK acts as an energy sensor, activating both fatty acid oxidation and mitochondria biogenesis, but also glycolysis [91]. Therefore, detrimental effects of AMPK reduction in ALS are likely to reflect accelerated hypermetabolism and energy deficit through inhibition of both catabolic pathways. In accord, we found a significant decrease in SOD1-G93A mice with respect to WT. Thus, AMPK activation can be of support. In this regard, metformin, a AMPK activator, was tested in a genetic model of the disease and found to be beneficial, improving neurologic phenotype in C9orf72 transgenic mice [92,93].

Recent findings have proposed a beneficial role of trimetazidine in preclinical studies in SOD1-G93A mice [94]. This is a metabolic modulator that inhibits the long-chain mitochondrial 3-ketoacyl coenzyme A thiolase (ACAA2), an enzyme responsible for the oxidation of long-chain fatty acids [95]. It was found to inhibit β-oxidation and fatty acid uptake, improving glucose metabolism [96]. Additionally, it increases mitochondrial protein levels and energy metabolism, and stimulates myogenesis, muscle strength, and oxidative metabolism in muscles, improving NMJ and neuromuscular communication [97]. Those effects extend survival of SOD1-G93A mice. Additionally, ranolazine, an FDA-approved inhibitor of fatty acid β-oxidation, favors the glycolytic process and led to an increase of ATP level in SOD1-G93A mice, and this effect correlates with a temporary recovery of the pathological phenotype [98]. Recently, niclosamide was proposed to be useful in preclinical trials. This drug interferes with different markers of the disease, such as mTOR, STAT3, and NF-κB. Thus, it reduces inflammation and aggregates formation in skeletal muscle, and displays beneficial effects also on muscle atrophy, by promoting regeneration [99]. It has been found that mutations of SOD1 induce upregulation of c-Abl, an apoptosis-related gene, and a decrease of cell viability. The expression of c-Abl was found to be increased in spinal cord from sporadic ALS patients [100]. Thus, the administration of dasatinib [101], a c-Abl inhibitor, was able to inactivate caspase-3, to inhibit cytotoxicity and to improve innervation at NMJ and survival of SOD1-G93A mice [100].

Many studies have focused on the alterations of neuronal and muscular excitability due to abnormalities in axonal sodium (Na^+^) and potassium (K^+^) conductance [102]. It is widely acknowledged that excitotoxicity importantly contributes to ALS by promoting a neurodegenerative cascade via Ca^2+^−mediated processes [103]. Accordingly, the voltage-sensitive Na+ channel blockers, such as mexiletine, have been tested to reduce hyperexcitability, promote membrane stabilization, and control ALS symptoms [72]. Mexiletine was tested in spinal cord cultured cells exposed to a medium derived from astrocytes expressing mutant SOD1-G93A and was found to reduce hyperexcitability, restore basal calcium transients, and prevent motoneuron death [104]. Thus, mexiletine was tested in clinical trials for muscle cramps. Additionally, retigabine, a K+ channels activator indicated as anti-convulsant, was proposed as a regulator of excitability, as well as in the reduction of reactive oxygen species generation [105]. In this regard, it is important to underline that riluzole, the most effective drug used in ALS, acts by inhibiting excitability. This demonstrates the need to improve this pathological aspect.

Our preclinical studies on MLC/SOD1-G93A animals reveal an important role of acetazolamide. Acetazolamide, a carbonic anhydrase inhibitor indicated for excitability disorders, when applied in-vitro, restored the ClC-1 chloride channel activity and sarcolemma hyperexcitability in MLC/SOD1-G93A mice [22]. Acetazolamide was already found to beneficially improve ClC-1 function in myotonia congenita through voltage-dependent regulation of the channel [19,106,107,108]. In support of the need to maintain chloride channel function, the application of chelerythrine, a PKC blocker was found to ameliorate the resting chloride conductance (gCl) in ALS mice, suggesting an important role of PKC-theta in the pathology [25,26,109,110].

Importantly, the benefit of physical activity (mild to moderate) on motor neuron loss and muscle atrophy has been documented in ALS [111,112]. Thus, it could be considered as a possible therapeutic intervention to delay muscle degeneration and to preserve NMJ integrity. Indeed, physical activity can promote myofiber regeneration by activating satellite cells. As a result, an improvement of metabolism and mitochondrial biogenesis can be possible in skeletal muscle [113]. An antioxidant effect and amelioration of GLUT4 glucose transporter expression was also demonstrated. Different studies on animal models have showed that exercises had beneficial effects in neurodegenerative diseases. For instance, swim training affects Akt signaling and ameliorates loss of skeletal muscle mass in a mouse model of ALS [114]. Moreover, myokines produced by skeletal muscle during exercise (i.e., BDNF and irisin) are thought to be beneficial through a variety of regulatory mechanisms, including cell survival, neurogenesis, improvement of neuroinflammation, protein degradation, and oxidative stress regulation. To date, only a few myokines have been investigated for their effects. Therefore, due to the possibility of these compounds to control many aspects of the disease, future studies are needed to better explore the role of myokines in ALS. In accord, in SOD1-G93A and MLC/SOD1-G93A mice, we previously found a decreased expression of irisin. This decrease may disturb muscle-nerve connection, suggesting its possible role in therapy. Thus, we focus our attention on irisin with the aim to restore muscle-nerve crosstalk and inflammatory response, and promising studies are in progress. Irisin is a recently discovered hormone released from skeletal muscle during exercise and is also considered as a crucial therapeutic agent in a wide variety of metabolic diseases [115]. Since irisin efficiently triggers metabolism and mitochondrial biogenesis in myocytes, it gained attention in the field of neuromuscular diseases. Irisin was found to contrast denervation and oxidative stress [27,116], suggesting a beneficial role in ALS. Recent studies showed that irisin expression increased in patients affected by metabolic disorders and cancer cachexia [117]. Moreover, it was found increased in blood of ALS patients with great disability [118]. Conversely, expression of irisin was reduced in patients with type 2 diabetes and non-alcoholic fatty liver disease. Most recent studies have demonstrated that serum irisin concentration was reduced in patients with breast cancer and exerts inhibitory effect on malignant breast cancer cells [117]. In this regard, further study of these pathways may be useful to assess the role of this myokine in a situation of inactivity such as ALS. Preclinical studies are required to examine the efficacy of these compounds before proposing clinical trials. These modulators can be useful also as a supportive therapy. In conclusion, drugs targeting skeletal muscle can be a new field of interest in ALS therapy and to ameliorate respiratory function.

**Table 2:** Preclinical studies in ALS models. In vivo and in vitro effects of different pharmacological compounds at pre-synaptic and post-synaptic level.

Administered Compound	Animal Model/In Vitro Model	Effects on Pre-Synaptic Target (MN, NMJ)	Effects on Post-Synaptic Target (Skeletal Muscle)	Effects on Survival	References
GDNF	SOD1-G93A rats	Amelioration of denervation	Lower rate of motor dysfunction	Increase of survival	[81]
	SOD1-G93A mouse model	Reduced rate of denervation and increased survival of spinal MNs	Improvement of locomotor performance	Increased life span by 17 days	[82]
VEGF	SOD1-G93A mouse model	Protection of spinal and brainstem motor neurons, increase of vascularization	Amelioration of locomotor performance	Increase of life expectancy	[84]
IGF1	SOD1-G93A mouse model	NMJ stabilization, reduced inflammation in the spinal cord, enhanced motor neuronal survival	Reduction of muscle atrophy	Increase of life expectancy	[14,87]
metformin	C9orf72 ALS/FTD mouse	Improvement of neurological phenotype	-	-	[93]
trimetazidine	SOD1-G93A mouse model	Prevention of NMJ dismantlement, attenuation of motor neuron loss and functional decline, reduction of neuroinflammation	Stimulation of energy metabolism, myogenesis, muscle strength and oxidative metabolism	Extension of survival	[94]
ranolazine	SOD1-G93A mouse model	-	marked increase in muscle strength and function	fail	[98]
niclosamide	ALS-FUS mice	Amelioration of axonal impairment	beneficial effects on muscle atrophy, increase of muscle regeneration and reduction of fibrosis.	-	[99]
dasatinib	SOD1-G93A mouse model	Improvement of the innervation status	Partial recovery of motor dysfunction	Improvement of survival	[100]
mexiletine	SOD1-G93A cultured cells	Prevention of MN death	-	-	[104]
retigabine	In vitro model of ALS	Reduction of MN excitability and death	-	-	[105]
PKC inhibitor	In vitro model of ALS (SOD1-G93A)	Prevention of NMJ dismantlement	Amelioration of muscle function	-	[25]
acetazolamide	In vitro model of ALS (SOD1-G93A)	-	Amelioration of muscle function	-	[22]

## 5. Conclusions

New evidences have highlighted the role of skeletal muscle in ALS etiopathogenesis. Indeed, NMJ dismantlement and muscle atrophy are early events in ALS and precede denervation, suggesting skeletal muscle as an important player in this pathology. Here, we have shown different drugs employed in preclinical and clinical studies that have targeted skeletal muscle with the aim to evidence possible therapeutic interventions that can improve its function and protect motor neurons. For instance, skeletal muscle ion channels are important for its function and can be determinant of ALS pathogenesis and target of drugs. We found involvement of the skeletal muscle chloride channel, ClC-1, a channel already observed to be involved in muscle channelopathies [119,120,121,122,123] and other neurodegenerative disorders [124]. This channel is affected during muscle atrophy [125,126], modified by oxidative stress [127] and growth factors changes [47,128], and its functional characterization links a reduction of ClC-1 activity to an abnormal sarcolemma hyperexcitability. Thus, skeletal muscle function can be ameliorated by the action of regenerative molecules that are able to restore the expression of pivotal genes and proteins, such as chloride channels. These important pro-myogenic factors can support skeletal muscle by mediating the effect of exercise and by sustaining myogenesis and neurogenesis. They can promote muscle hypertrophy and improve muscle strength, and ameliorate metabolism and energy production [27]. These effects can be of importance to slow down the progression of the disease.

## Data Availability

Not applicable.
